# Selected clinical approaches for the treatment of classic Class II, division 1 malocclusion

**DOI:** 10.1590/2177-6709.31.3.e262655.oar

**Published:** 2026-08-07

**Authors:** Orlando Motohiro TANAKA, Gil Guilherme GASPARELLO, Matheus Melo PITHON, Oscar Mario ANTELO, Renato Parsekian MARTINS

**Affiliations:** 1 Pontifícia Universidade Católica do Paraná. Escola de Medicina e Ciência da Vida (Curitiba/PR, Brazil).; 2 University of Oulu, Research Unit of Population Health (Oulu, Finland).; 3 State University of Southwest Bahia (Jequié/BA, Brazil).; 4 Christian University of Bolivia, School of Dentistry, Department of Orthodontics (Santa Cruz de la Sierra, Bolivia).; 5 Private practice (Araraquara/SP, Brazil).

**Keywords:** Class II malocclusion, Orthodontic treatment planning, Temporary anchorage devices, Tooth extraction, Orthognathic surgery, Diagnosis, Biomechanics, Má oclusão de Classe II, Planejamento do tratamento ortodôntico, Dispositivos de ancoragem temporária, Extração dentária, Cirurgia ortognática, Diagnóstico, Biomecânica

## Abstract

**Introduction:**

Class II, division 1 malocclusion is the most common sagittal discrepancy, marked by distal positioning of the lower teeth, upper incisor protrusion, maxillary constriction, and facial profile imbalance. Its multifactorial etiology-skeletal, dental, and functional - results in diverse clinical presentations and treatment approaches.

**Objective:**

This article aims to present a series of clinical approaches for the management of classic Class II, division 1 malocclusion, underlining how different treatment modalities -selected according to individual patient characteristics- can lead to effective outcomes.

**Considerations:**

Treatment strategies for Class II, division 1 malocclusion include growth modification, headgear therapy, maxillary molar distalization with temporary anchorage devices (TADs), premolar extractions, clear aligner therapy, and orthodontic-surgical approaches for severe skeletal cases. Advances in biomechanics and skeletal anchorage have expanded non-extraction and less invasive options. However, careful case selection remains essential. Diagnosis must assess skeletal, dental, functional, and psychosocial factors, with treatment choices balancing esthetics, biomechanics, stability, and patient cooperation, customized to individual needs

## RESUMO

**Introdução:**

A má oclusão de Classe II, divisão 1, é a discrepância sagital mais comum, caracterizada pelo posicionamento distal dos dentes inferiores, protrusão dos incisivos superiores, constrição maxilar e desequilíbrio do perfil facial. Sua etiologia multifatorial - esquelética, dentária e funcional - resulta em uma diversidade de combinações clínicas e alternativas de tratamento.

**Objetivo:**

Este artigo teve como objetivo apresentar uma série de alternativas clínicas para o manejo da má oclusão de Classe II, divisão 1, clássica, destacando como diferentes modalidades de tratamento, selecionadas de acordo com as características individuais de cada paciente, podem levar a resultados eficazes.

**Considerações:**

As estratégias de tratamento para a má oclusão de Classe II, divisão 1, incluem modificação do crescimento, uso de aparelho extrabucal, distalização dos molares superiores com dispositivos de ancoragem temporária (DATs), extrações de pré-molares, alinhadores transparentes e abordagens ortodôntico-cirúrgicas para casos esqueléticos severos. Os avanços em biomecânica e ancoragem esquelética ampliaram as alternativas sem exodontias e menos invasivas. No entanto, a seleção criteriosa dos casos permanece essencial. O diagnóstico deve avaliar fatores esqueléticos, dentários, funcionais e psicossociais, com escolhas terapêuticas que equilibrem estética, biomecânica, estabilidade e cooperação do paciente, personalizadas às necessidades individuais.

## INTRODUCTION

Class II division 1 malocclusion is defined by abnormal mesiodistal relations between dental arches; all the lower teeth occluding distal relative to normal position, producing very marked disharmony in the incisive region and in the facial lines, and is characterized by a narrowing of the upper arch, lengthened and protruding upper incisors, accompanied by abnormal function of the lips and some form of nasal obstruction and mouth-breathing.^1^

Representing the most prevalent sagittal discrepancy, its origin may be dental, skeletal, or mixed, involving mandibular deficiency, maxillary excess, or both.^2^ The multifactorial etiology includes skeletal, dental, and functional components, contributing to wide clinical variability and a correspondingly diverse array of therapeutic approaches.^3^

Traditional management includes extraoral appliances, dental extractions, and, in more severe cases, orthognathic surgery. However, advances in orthodontic biomechanics and skeletal anchorage systems have considerably expanded non-extraction treatment alternatives. Maxillary molar distalization with TADs has emerged as an effective strategy for many cases, reducing the need for extractions.^4^

Complex cases, such as those involving dentoalveolar bimaxillary protrusion, severe space discrepancies, or temporomandibular joint (TMJ) dysfunction, still necessitate carefully planned extractions. Such biomechanically driven extraction protocols can enhance facial aesthetics, occlusal stability, and TMJ symptom relief.^5^

Clear aligners represent an increasingly popular treatment modality, valued for aesthetics and patient comfort.^6^ Despite their technological evolution and improved control over tooth movement, biomechanical limitations remain, particularly in extraction cases and complex three-dimensional corrections.^6^ ^,^ ^7^ Systematic reviews confirm their effectiveness in mild-to-moderate cases, but suggest caution in more severe Class II cases.^8^

For patients with severe skeletal discrepancies not suitable to conventional orthodontics, combined orthodontic-surgical treatments remain the standard, especially in adults. Mandibular advancement alone is often sufficient to address mandibular retrognathia, but in selected cases, superior maxillary impaction or bimaxillary surgery may be required to optimize occlusion and facial harmony.^9^

Treatment decisions often involve mandibular response modulation, upper arch extractions, or four-premolar extraction strategies. The present article presents ten representative clinical cases that illustrate this diversity in evidence-based management pathways for the classical Class II Division 1 malocclusion.

## DISCUSSION

Class II division 1 malocclusion is a multifactorial orthodontic condition with a broad range of treatment alternatives that must be carefully individualized, based on skeletal, dental, functional, and psychosocial criteria. The clinical cases presented in this article illustrate a spectrum of available strategies, emphasizing the importance of technique selection according to case severity, patient age, facial profile, required cooperation, and technical expertise.

Two-phase non-extraction treatments with functional appliances have demonstrated favorable outcomes in young patients with mandibular growth potential. These approaches are effective for addressing primarily functional discrepancies and enhancing mandibular growth using devices such as the open elastic Klammt activator,^10^ ^,^ ^11^ as illustrated in Figure 1. However, the magnitude of the skeletal response remains limited, and dentoalveolar effects - such as lower incisor proclination - must be carefully considered.^12^

**Figure 1:**
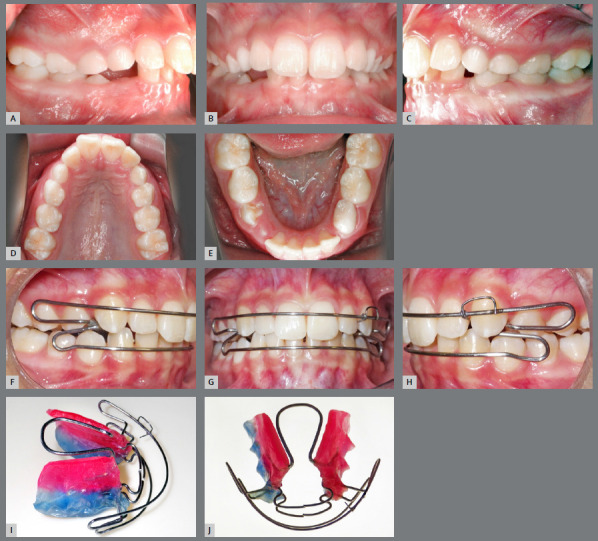
A-E) Class II division 1 malocclusion initially treated with a Klammt’s elastic open activator, followed by a corrective phase with fixed appliances. F-J) The placement of the Klammt open elastic activator involved an initial adaptation period ranging from two to four weeks. Following this, the patient was instructed to wear the appliance full-time, except during meals and sports activities. Follow-up appointments were scheduled every 15 days, with monthly activations of approximately 0.25 mm using a bird-beak plier. After 16 months of active interceptive treatment, the results showed molar and canine relationships in Class I, with proper overjet and overbite.

[Fig f1a]

**Figure 1 f1a:**
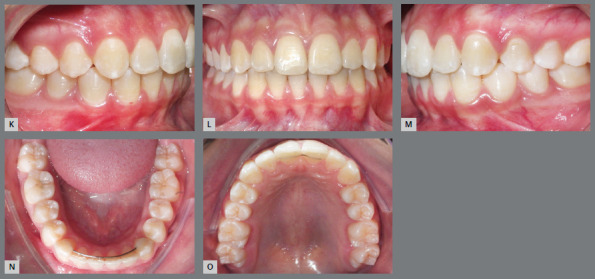
(continued): **K-O)** Occlusal and functional refinement was achieved using fixed appliances.

Mandibular growth response, when combined with Kloehn-type headgear, has shown efficacy in favorable growth pattern patients with high compliance,^13^ as shown in Figure 2. This appliance enables maxillary molar distalization and exerts orthopedic restriction on maxillary growth, being most effective in growing patients.^9^ However, results are highly dependent on skeletal age and patient cooperation, with reduced outcomes in low-compliance or older patients.^8^

**Figure 2:**
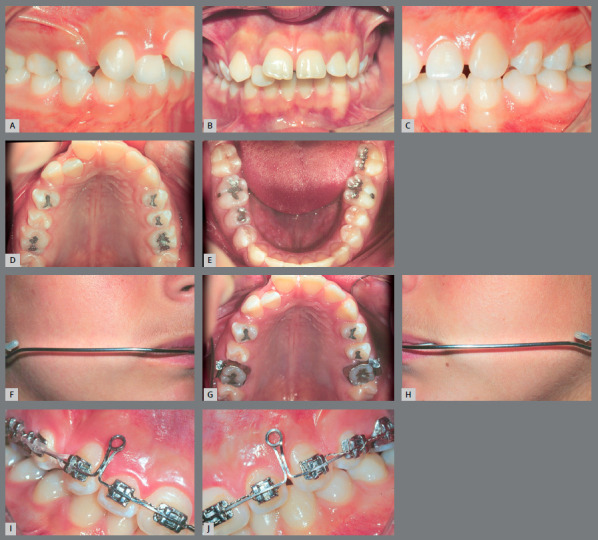
A-E) Class II division 1 malocclusion treated using Kloehn-type cervical headgear for distalization of maxillary molars and premolars. F-J) The Kloehn-type extraoral appliance was worn for 15 hours per day, effectively promoting distal movement of the maxillary molars and premolars. The diastemas created were consolidated between the canines and lateral incisors to enable anterior retraction of the maxillary incisors using helicoidal loop mechanics with a 0.019 × 0.026-in stainless steel archwire, with the headgear providing anchorage.

[Fig f2a]

**Figure 2 f2a:**
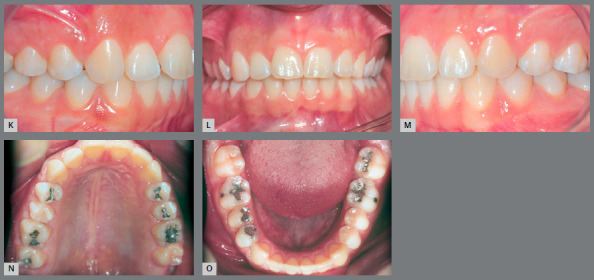
(continued): **K-O)** No mandibular appliances were used during treatment.

Although the extraoral appliance has been historically used as a standard or adjunctive approach in the treatment of Class II division 1 malocclusion,^13^ its indication remains relevant in contemporary practice, as neither the malocclusion nor its biomechanical effects have changed; rather, its current decline in use is mainly due to the lack of emphasis by instructors in continuing education programs.^14^

The introduction of TADs marked a significant advancement in orthodontic biomechanics, providing an efficient and esthetically acceptable alternative for non-extraction maxillary molar distalization with reduced patient compliance dependency^15^ (Fig. 3). 

**Figure 3:**
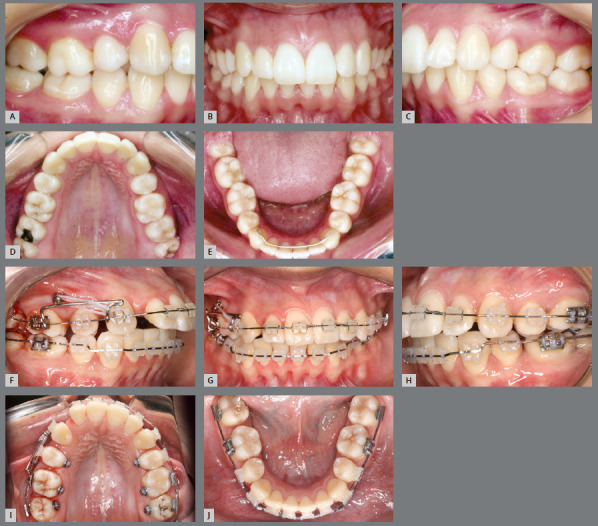
A-E) Class II division 1, subdivision with upper midline deviation to the left. F-J) Treatment involved extraction of the upper second molars and lower third molars. Right-side maxillary teeth were distalized using a sliding jig supported by TADs, while distalization was also performed on the left maxillary side and mandibular posteriors. Anterior teeth were retracted.

[Fig f3a]

**Figure 3 f3a:**
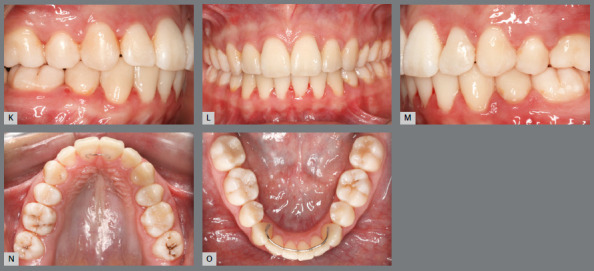
(continued): **K-O)** Finishing was done ensuring molar and canine key occlusion. Retention included a fixed retainer between the upper central incisors, and a canine-to-canine retainer in the mandible.

TADs have proven effective as adjuncts for upper molar distalization, providing stable and predictable outcomes while minimizing facial esthetic compromise during treatment,^4^ as demonstrated in Figure 4. 

**Figure 4:**
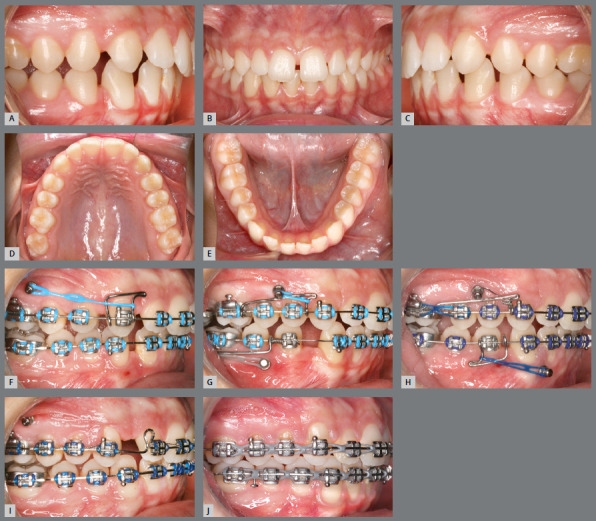
A-E) Class II division 1 malocclusion with upper and lower diastemas. F-J) Treatment involving maxillary distalization and mandibular mesialization using sliding jigs and mini-implants with intermaxillary elastics. Maxillary posterior teeth were distalized and mandibular posterior teeth, mesialized with the aid of sliding jigs and TADs, utilizing intermaxillary elastics for force application. Retraction of the upper anterior teeth was performed using teardrop loops.

[Fig f4a]

**Figure 4 f4a:**
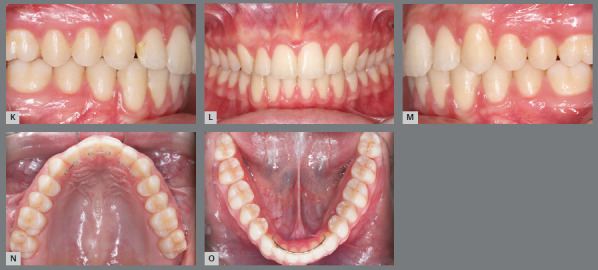
(continued): **K-O)** Finalization involved coordinated rectangular archwires, to achieve proper intercuspation and arch coordination. Retention consisted of a full-tooth bonded retainer in the maxilla and a canine-to-canine bonded retainer in the mandible. This biomechanically controlled approach reflects current trends, favoring skeletal anchorage for non-extraction correction of Class II malocclusions.

In cases with dentoalveolar discrepancies restricted to the maxilla, extraction of upper premolars has demonstrated an efficient strategy for upper incisor retraction and facial profile improvement without compromising functional occlusion^16^ (Fig. 5). For patients presenting with bimaxillary protrusion or severe crowding, the extraction of four premolars remains a time-tested approach for achieving balanced space distribution and comprehensive three-dimensional correction when appropriately planned and executed.^5^

**Figure 5:**
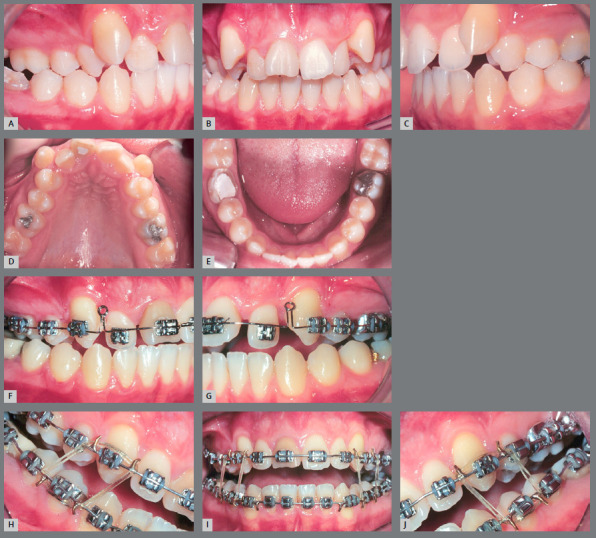
A-E) Class II, division 1 malocclusion. F-J) Extraction of the maxillary first premolars was performed. Initial retraction of the central incisors - excluding the lateral incisors - was carried out using a 0.016-in stainless steel archwire. Rectangular archwires and intermaxillary elastics with a Class II vector were subsequently used.

[Fig f5a]

**Figure 5 f5a:**
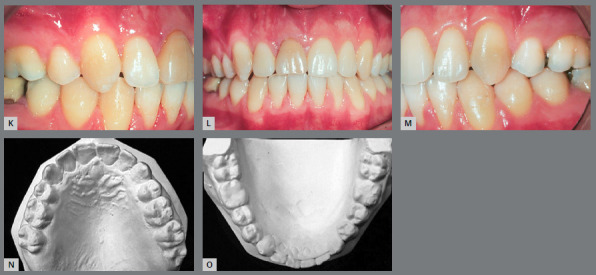
(continued): K-O) Finishing achieved in canine key occlusion and molars maintained in a Class II relationship.

Clear aligners were successfully employed in a mild-to-moderate case involving extractions, intermaxillary elastics, and TADs,^17^ as illustrated in Figure 6. Although clear aligners show limitations in managing complex movements such as root torque and rotations, they provide notable advantages regarding comfort and esthetics, and their indications should be carefully assessed on a case-by-case basis.^6^ ^,^ ^7^

**Figure 6:**
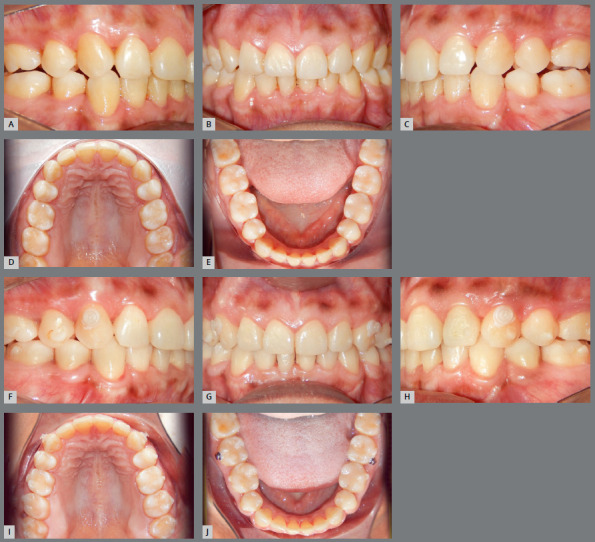
A-E) Class II division 1 malocclusion previous extractions of four first bicuspids and bilateral posterior crossbite. F-J) Orthodontic retreatment with four first bicuspid extraction and using clear aligners, with sequential upper arch distalization and Class II elastics. The first aligner set consisted of 49 upper and lower aligners, combining sequential maxillary distalization and Class II elastics. The second set included 47 upper aligners and 11 active lower aligners. Attachments were bonded to enhance movement predictability, combined with Class II elastics.

[Fig f6a]

**Figure 6 f6a:**
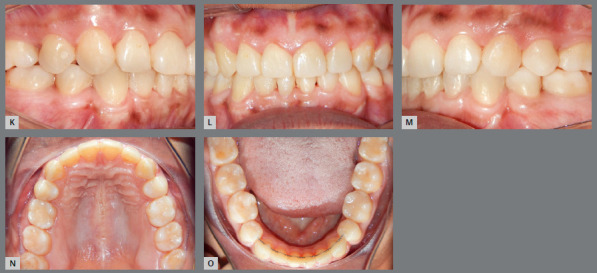
(continued): **K-O)** A good occlusal outcome was achieved.

For adult patients with severe skeletal discrepancies, orthognathic surgery was the only approach capable of fully correcting skeletal, dental, and facial disharmonies tridimensionally (Fig. 7). While mandibular advancement alone was effective in most cases, selected cases required bimaxillary surgery to achieve optimal functional and esthetic outcomes, with both procedures demonstrating high postoperative stability.^18^

**Figure 7:**
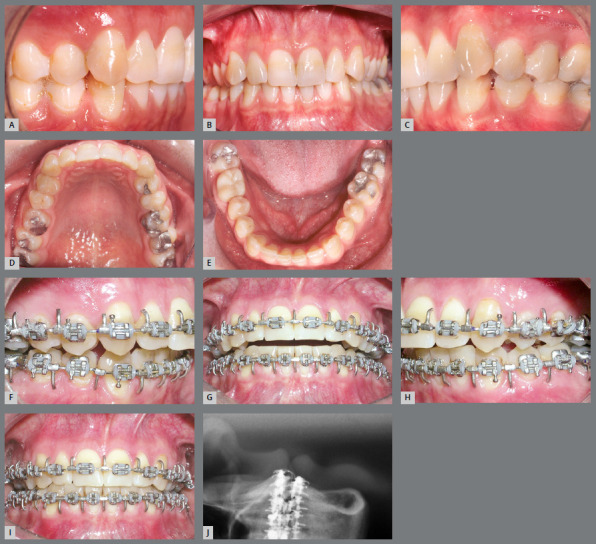
A-E) Class II division 2 malocclusion. F-J) Treatment involved the proclination of the maxillary incisors and uprighting of the mandibular incisors, optimized by the presence of a mild diastema in the lower arch, with the objective of creating sufficient overjet for mandibular advancement surgery.

[Fig f7a]

**Figure 7 f7a:**
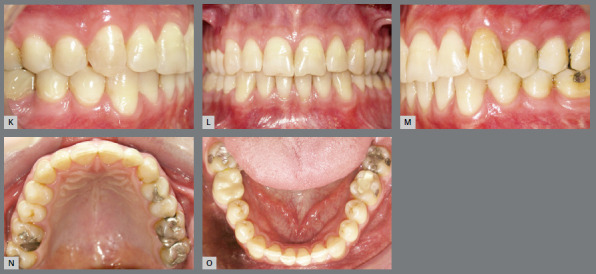
(continued): **K-O)** The patient’s expectations for both esthetics and function were successfully met.

In summary, the clinical cases presented in this article underscore that different treatment modalities are not mutually exclusive, but can be synergistically combined during treatment planning, provided that a complete and accurate diagnosis is conducted. Integrating orthopedic, mechanical, and esthetic strategies can optimize clinical outcomes, particularly in borderline or moderately complex cases in which a single-technique approach might be insufficient.

**Figure 8:**
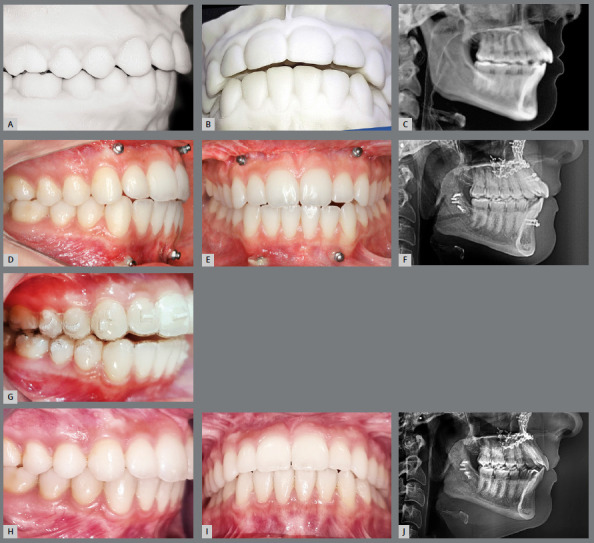
A-J) Skeletal Class II division 1 corrected with a surgery-first approach followed by clear aligner therapy. A surgery-first protocol was used to correct the skeletal discrepancy, followed by finishing and detailing with aligners.

For example, the combined use of TADs with clear aligners (Fig. 8) or with intermaxillary elastics can enhance biomechanical control in Class II cases with space limitations or increased anchorage demands. In selected cases, a hybrid treatment approach is recommended.^19^ Figure 9 illustrates the combination of a distalizer bonded from the upper first molars to the canines with lower molar buttons to facilitate full-time intermaxillary elastics. Following the improvement in sagittal relationships, upper arch aligner therapy is initiated to complete the correction and refinement phase. Similarly, two-phase functional orthopedic therapy can amplify early orthopedic effects, facilitating the fixed appliance phase and ensuring long-term stability during alignment and leveling.

**Figure 9:**
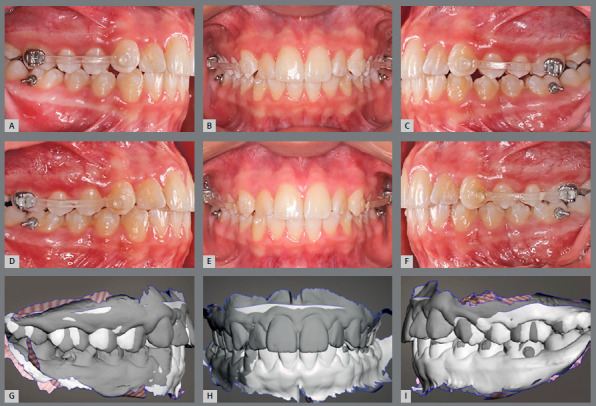
A-C) Intraoral frontal and lateral pictures of a patient with a Class II division 1 malocclusion treated with a Carriere-type distalizer (Iceram Distalizer, Orthometric, Marília, Brazil) and aligners. The distalizers were bonded to the maxillary first molars and canines. In the mandibular arch, molar buttons with hooks (Orthometric, Marília, Brazil) were bonded to the first molars for full-time wear of 1/4-in, 6-oz intermaxillary elastics. Simultaneously, treatment of the mandibular arch was initiated with aligners (E-Motion Aligners, Ortho E-Motion, Marília, Brazil), incorporating cutouts around the buttons. D-F) Intraoral frontal and lateral pictures after 40 days. Rapid correction was achieved, with marked improvement in the sagittal relationship. It should be noted that correction occurred unusually quickly in this patient and is not representative of the rate typically observed in clinical practice. The patient was instructed to continue elastic wear for an additional month to obtain overcorrection. G-I) Superimposition of the patient’s digital scans obtained at the time of panels A-C and D-F, depicting the effects of the distalizer. In the maxillary arch, molar derotation and distalization were observed, along with distal movement of the premolars and canines and slight incisor retraction. In the mandibular arch, mild anterior projection of the dental arch was observed.

[Fig f9a]

**Figure 9 f9a:**
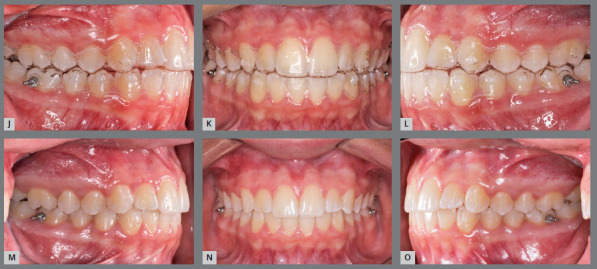
(continued): **J-L)** Intraoral frontal and lateral pictures at the start of maxillary aligner therapy. It is important to note that, in these images, the aligners were being worn and prevented complete intercuspation, creating the appearance of an incomplete anteroposterior correction. **M-O)** Completion of aligner therapy after six months of treatment. No Class II elastics were required during the subsequent aligner phase and therefore were not used.

The rational integration of these modalities, guided by scientific evidence and adapted to each patient’s individual profile, reflects the evolution of contemporary management for the classic Class II division 1 malocclusion, enabling more personalized, efficient, and predictable treatments.^20^ Each approach has distinct indications and limitations, and clinical decision-making must carefully balance esthetics, biomechanics, and long-term stability in a patient-centered framework. 

Ultimately, decisions should balance esthetics, biomechanics, and long-term stability, adapted to individual patient profiles. Avoiding necessary extractions when indicated can result in under-treatment and patient dissatisfaction. Correct management of midline discrepancies in Class II subdivision cases requires careful etiology assessment and patient-involved decision-making, with extractions considered when needed,^21^ as shown in Figure 10.

**Figure 10:**
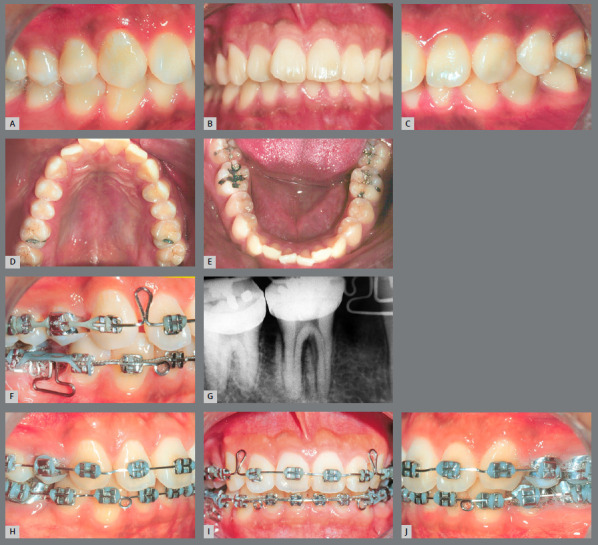
A-E) Class II division 1, subdivision with lower midline shift to the right. F-J) Treatment involved extraction of the upper first premolars, the lower left first premolar, and the lower right second premolar. The latter was extracted to facilitate anchorage loss for molar mesialization. Mechanics included T-loops and intermaxillary elastics for bodily molar movement (confirmed radiographically). However, mesialization of the lower first molar proved difficult, suggesting that avoiding the second premolar extraction on the lower right side could have allowed case completion with molars in Class II on that side.

[Fig f10a]

**Figure 10 f10a:**
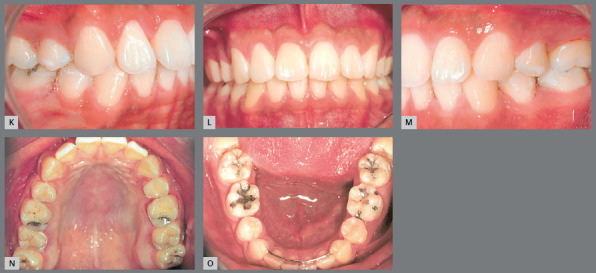
(continued): **K-O)** A good occlusal outcome was achieved.

Malocclusions remain a persistent clinical challenge, but the management of classic Class II division 1 has evolved with multiple adjunctive innovations over recent decades.^22^ The orthodontic “A” - representing appliances in the ABCDE... sequence - now reflects a broader understanding that esthetics, function, and stability must be guided by thorough and sensible diagnostic protocols.^23^

## FINAL CONSIDERATIONS

The management of classic Class II, division 1 malocclusion requires individualized treatment planning based on thorough diagnostic evaluation. The cases presented in this article highlight that no single approach fits all patients. Successful outcomes depend on selecting the most appropriate technique - or combination of techniques -, considering skeletal and dental characteristics, growth potential, facial esthetics, patient cooperation, and long-term stability. The integration of traditional and contemporary resources, guided by evidence-based decision-making, allows clinicians to deliver more predictable and patient-centered results.

## Data Availability

All data generated or analyzed during this study are included in this published article.
